# Acute Dystonia With Rhabdomyolysis Induced by Paliperidone Palmitate: A Rare Adverse Effect

**DOI:** 10.7759/cureus.28771

**Published:** 2022-09-04

**Authors:** Nishant Allena, Sai Doppalapudi, Sneha Khanal, Steven Tank, Rabih Nasr

**Affiliations:** 1 Internal Medicine, BronxCare Health System, Bronx, USA; 2 Nephrology, BronxCare Health System, Bronx, USA

**Keywords:** invega sustenna, extrapyramidal side effects, rhabdomyolysis, cervical dystonia, paliperidone palmitate

## Abstract

Antipsychotic medications have been well-established to potentially cause extrapyramidal side effects (EPS) including hyperkinesia, tremor, dyskinesia, dystonia, and parkinsonism. Rhabdomyolysis secondary to extrapyramidal symptoms in patients under antipsychotics is a relatively rare presentation to be observed in patients.

In this report, we present a 64-year-old female with rhabdomyolysis following a once-monthly injection of long-acting injectable (LAI) paliperidone palmitate (Invega Sustenna). The patient developed extrapyramidal symptoms one day after the paliperidone injection. She presented with acute dystonia in the form of antecollis, without any evidence of generalized myalgia or kidney involvement. Laboratory investigations demonstrated a creatine kinase (CK) level of 3239 unit/L on admission. The patient’s symptoms were resolved after the administration of benztropine and cyclobenzaprine and CK levels improved after IV hydration.

A high index of suspicion in the investigation of rhabdomyolysis for patients presenting with extrapyramidal symptoms being treated with long-acting injectable antipsychotics leads to prompt diagnosis, early treatment, and reduction in renal and cardiac toxicities in the aforementioned population.

## Introduction

Drug-induced movement disorders, known as extrapyramidal side effects (EPS), are among the common adverse drug effects experienced by patients taking dopamine-receptor blocking agents [1]. Long-acting injectable (LAI) paliperidone has the risk of parkinsonism (7%), akathisia (4%), and dyskinesia (3%) [2]. There have been studies that inculpate antipsychotics to cause severe constipation as an adverse effect as well [3]. Other notable side effects of antipsychotics include diabetes, weight gain, and hyperlipidemia [4]. Whether acute dystonia occurs as a result of the use of antipsychotic drugs depends mainly on the presence of risk factors such as affective disorders. Therefore, the prevalence varies widely from 2% to 90%. Rhabdomyolysis induced by acute dystonia is a relatively rare adverse effect that can ensue either from a combination of risk factors or when musculoskeletal trauma from one EPS like acute dystonia is significant enough. 

Here we describe a case of acute dystonia causing rhabdomyolysis induced by an LAI antipsychotic, paliperidone palmitate, which was administered as monthly injections. 

## Case presentation

A 64-year-old female was admitted to our institute with the complaint of sudden flexion of her neck associated with pain for four days. Her medical history was remarkable for a history of bipolar disorder, schizoaffective disorder, hypertension, and diabetes. She was being treated with lithium 450 mg twice a day, Invega Sustenna 234 mg/1.5 mL intramuscular suspension extended release intramuscularly every four weeks, sertraline 25 mg once a day, and trazodone 50 mg once a day. The patient’s complaints started a day after receiving an IM injection of Invega Sustenna.

Laboratory testing on presentation reported hemoglobin of 10.7 g/dL, serum creatinine of 0.8 mg/dL, serum potassium of 3.1 mEq/L, serum sodium of 139 mEq/L, serum magnesium of 2.4 mg/dL, serum phosphorus of 3.3 mg/dL and serum creatine kinase (CK) level of 3239 unit/L. Urine toxicology was positive for cannabinoids and the estimated GFR was 85.42 mL/min/1.73m2. The patient's laboratory values are presented in Table 1. 

**Table 1:** Laboratory values during the course of hospital stay

Laboratory Parameter	Admission Day	Day 2	Day 3	Reference Range
Hemoglobin (g/dL)	10.7	11.6	11.2	12-16 g/dL	
Serum Creatinine (mg/dL)	0.8	0.7	0.8	0.5-1.5 mg/dL
Serum Potassium (mEq/L)	3.1	3.7	3.7	3.5-5 mEq/L
Serum Sodium (mEq/L)	139	143	141	135-145 mEq/L
Serum Phosphorus (mg/dL)	3.3	3.4	3.3	2.5-4.5 mg/dL
Serum Magnesium (mg/dL)	2.4	2.3	2.5	1.5-2.7 mg/dL
Serum Creatine Kinase (CK) (unit/L)	3239	1772	1119	20-200 unit/L

The patient was hence admitted to the General Medicine floor for management of rhabdomyolysis. Her physical examination was noteworthy of antecollis with marked tenderness over the sternocleidomastoid muscles on palpation and exacerbation of pain on passive range of motion. Given the timing of her symptoms and administration of Invega a day prior, acute dystonia was suspected as the extrapyramidal side effect and the patient was started on benztropine 1 mg orally every 12 hours as per psychiatry recommendations. She was also treated with lactated ringers at 100 cc/hr and cyclobenzaprine 5 mg orally was added to her medication regimen to help ease the pain.

The CK levels were followed daily and showed a down-trending pattern. Furthermore, the patient experienced significant improvement in the range of motion of her neck in addition to pain relief. She was then discharged on benztropine with a psychiatry outpatient follow-up for further management of antipsychotics.

## Discussion

Dystonia can be defined as a movement disorder characterized by its intermittent or sustained muscle contractions that lead to abnormal, often repetitive, movements or postures or both. Dystonia can range from focal minor contractions affecting only an individual muscle group or severe and disabling movements affecting multiple muscle groups. There are reported to be around 50,000 cases in the United States diagnosed with dystonia although the number could be higher because many cases go undiagnosed [5,6].

Dystonia is thought to be initiated by voluntary action and it is associated with overflow muscle activation. Acute dystonic reactions are hypothesized to be the result of a dopaminergic-cholinergic imbalance in the basal ganglia [7]. Some of the common causative agents of acute dystonic reactions are antipsychotic and antiemetic agents. Antipsychotic agents with a dopamine-blocking mechanism are commonly used to treat acute psychosis, acute agitation, bipolar mania, and many other psychiatric conditions. All antipsychotics carry a risk of causing acute dystonic reactions with first-generation antipsychotics associated with a higher risk of acute dystonic reactions. Second-generation antipsychotics have a lower risk of dystonic reactions, hypothesized to be due to the rapid dissociation of the drugs from the D2 receptor sites [8].

The exact mechanism of rhabdomyolysis induced by antipsychotics remains relatively unknown but there have been hypotheses that the antagonism of 5HT2A receptors is responsible. It is believed that antagonism of 5HT2A (serotonin 2A) receptors leads to the compromise of glucose uptake leading to the increased permeability of the sarcolemma to CK [9].

Paliperidone palmitate is a second-generation antipsychotic, which is an active metabolite of risperidone [10]. It is used for the treatment of schizophrenia and schizoaffective disorders. A post hoc analysis associated paliperidone LAI with the side effects of tardive dyskinesia, dystonia, and parkinsonism [2].

Acute dystonia constitutes around 1% of the EPS caused by paliperidone. So far, the antipsychotics that have been labeled to cause rhabdomyolysis include aripiprazole, clozapine, and ziprasidone. However, there has not been much literature published involving newer antipsychotics like paliperidone. Our search yielded two case reports involving paliperidone as the cause of rhabdomyolysis [11,12]. 

The treatment for acute dystonia revolves around balancing the disrupted dopaminergic and cholinergic balance in the basal ganglia and discontinuation of the offending agent. The most commonly used drugs are diphenhydramine and benztropine. Intravenous diphenhydramine is used for its anticholinergic effect and central nervous system (CNS) penetration and benzodiazepines may be considered for patients that fail to respond completely to anticholinergic therapy [13,14].

With this case, we would like to emphasize the need to rule out rhabdomyolysis in patients presenting with acute dystonic reactions even with newer generation antipsychotics like paliperidone.

## Conclusions

The presentation of rhabdomyolysis is varied, ranging from being completely asymptomatic to having muscle tenderness and weakness, which could be masked in cases of acute dystonia. The asymptomatic nature of rhabdomyolysis until a severe disease process ensues may be a reason for this unfathomable side effect to be under-reported. With not much literature involving newer-generation antipsychotics and rhabdomyolysis, further studies are required to assess the incidence of rhabdomyolysis manifesting with extrapyramidal symptoms. We recommend physicians monitor for rhabdomyolysis with CK levels in patients presenting with acute dystonic reactions even with newer-generation antipsychotics like paliperidone palmitate, as a prompt diagnosis and early treatment can prevent renal injury and life-threatening cardiac dysrhythmias.
